# Predictive Value of Residual SYNTAX Score II for Patients With Complex Coronary Disease and Chronic Renal Insufficiency After Percutaneous Coronary Intervention

**DOI:** 10.31083/RCM26962

**Published:** 2025-05-27

**Authors:** Shuaiyong Zhang, Yumeng Lei, Jingfu Chen, Youcheng Wang, Huanting Liu, Nan Guo, Yunfei Wang, Xufen Cao, Liqiu Yan

**Affiliations:** ^1^Department of Cardiology, The Affiliated Dongguan Songshan Lake Central Hospital, Guangdong Medical University, 523326 Dongguan, Guangdong, China; ^2^Department of Cardiology, Cangzhou Central Hospital, Hebei Medical University, 061001 Cangzhou, Hebei, China

**Keywords:** residual SYNTAX score II, coronary artery disease, chronic renal insufficiency, percutaneous coronary intervention

## Abstract

**Background:**

The primary objective of this research was to determine the predictive value of the residual SYNTAX (Synergy Between Percutaneous Coronary Intervention With Taxus and Cardiac Surgery) score II (rSS-II) for long-term outcomes in individuals with complex coronary artery disease (CAD) and chronic renal insufficiency (CRI) who have undergone percutaneous coronary intervention (PCI).

**Methods:**

A total of 1161 consecutive patients with complex CAD and CRI after PCI were retrospectively recruited from Cangzhou Central Hospital affiliated with Hebei Medical University between January 2014 and September 2017. The patients were stratified into three categories based on rSS-II tertiles: low rSS-II (n = 388), medium rSS-II (n = 389), and high rSS-II (n = 384). The primary endpoints were all-cause mortality (ACM) and cardiac mortality (CM), while the secondary endpoint was major adverse cardiovascular and cerebrovascular events (MACCEs), which included ACM, myocardial infarction, stroke, or unplanned revascularization. The discrimination, calibration, and clinical utility of the rSS-II for predicting long-term outcomes were examined.

**Results:**

The median follow-up period was 37 months (19 to 61 months). The Kaplan–Meier estimate rates of ACM (2.4% vs. 5.9% vs. 13.9%;* p* < 0.001) and CM (1.9% vs. 2.8% vs. 9.2%; *p* < 0.001) revealed significant differences among the three categories. Multivariate Cox regression analysis demonstrated that the rSS-II could independently predict ACM (hazard ratio: 1.08, 95% confidence interval: 1.04–1.12; *p* < 0.001) and CM (hazard ratio: 1.07, 95% confidence interval: 1.02–1.12; *p* = 0.009). The rSS-II performed satisfactorily in both discrimination (area under the curve for ACM and CM was 0.710 and 0.728, respectively) and calibration (Greenwood–Nam–D’ Agostino goodness-of-fit test for long-term outcomes; *p* > 0.05 for all). Additionally, decision curve analysis showed that the rSS-II had a high net benefit for long-term outcomes over threshold probabilities, indicating its superiority in daily practice.

**Conclusions:**

The rSS-II is beneficial for predicting and stratifying the risk of long-term outcomes in individuals with complex CAD and CRI who have undergone PCI.

## 1. Introduction

Previous studies have validated the favorable effect of complete revascularization (CR) on the prognoses of patients with complex coronary artery disease (CAD) [1, 2, 3]. The COMPLETE trial, which compared CR versus culprit-only revascularization strategies for treating multivessel disease after early percutaneous coronary intervention (PCI) for ST-elevation myocardial infarction (STEMI), demonstrated that CR was better than culprit-lesion-only PCI in lowering the risk of adverse cardiovascular events in individuals affected by STEMI and multivessel CAD [4]. Additionally, renal function impairment has been associated with rapid plaque progression, potentially contributing to the vulnerability of coronary plaques and increasing the risk of CAD [5, 6]. Furthermore, the Grand Drug-Eluting Stent (Grand-DES) registry, which included five multicenter national Korean registries, reported that CR leads to improved clinical results in individuals with chronic kidney disease (CKD) compared to incomplete revascularization (IR) [7]. Therefore, the complexity and severity of coronary disease and renal function determine the PCI strategy for CR or IR in clinical settings. Hence, risk stratification and evaluation of residual disease should be performed for the long-term prognosis of individuals with complex CAD and chronic renal insufficiency (CRI) after PCI.

The development of the SYNTAX (Synergy Between Percutaneous Coronary Intervention With Taxus and Cardiac Surgery) score (SS), also known as the pre-PCI SS, was aimed at evaluating the anatomic complexity of angiographic stenoses and facilitating decision-making for the optimal revascularization approach in individuals with complex CAD [8]. However, previous research has revealed the association of SS with mortality and adverse cardiovascular events in acute coronary syndrome (ACS) [9, 10]. Despite its usefulness, the lack of clinical variables in SS has been identified as a limitation by European and United States revascularization guidelines [11, 12]. To overcome these limitations, the SYNTAX score II (SS-II) was developed, which combines anatomical factors (SS and unprotected left main coronary artery (ULMCA) disease) with six clinical variables (age, creatinine clearance, left ventricular ejection fraction (LVEF), sex, chronic obstructive pulmonary disease (COPD), and peripheral vascular disease). Compared to SS, SS-II has shown a gradually increasing predictive value for long-term mortality in individuals with complex CAD undergoing PCI [13]. Head* et al*. [1] reported that IR was associated with unfavorable clinical outcomes after PCI in a 3-year SYNTAX trial, and the post-PCI SYNTAX score, such as the residual SYNTAX score (rSS), was developed to evaluate the predictive value of IR after PCI in the Acute Catheterization and Urgent Intervention Triage Strategy (ACUITY) trial, quantifying the residual burden of anatomical coronary disease [2]. The high-rSS group was associated with an elevated risk of clinical comorbidity and a disease characterized by more anatomical complications compared to low-rSS groups [3]. Additionally, previous studies have shown that rSS is relevant to poorer clinical outcomes both during the in-hospital period and the subsequent long-term period after discharge [14, 15]. Recently, the post-PCI SS-II, such as the residual SS-II (rSS-II), has been validated for predicting long-term outcomes in individuals with ACS [16, 17]. However, no prior studies have evaluated the predictive value of rSS-II in patients with complex CAD and CRI. Therefore, the main objective of this research was to validate the prognostic value of rSS-II in individuals with complex CAD and CRI after PCI.

## 2. Materials and Methods

### 2.1 Study Subjects and Design

**Fig. 1.**
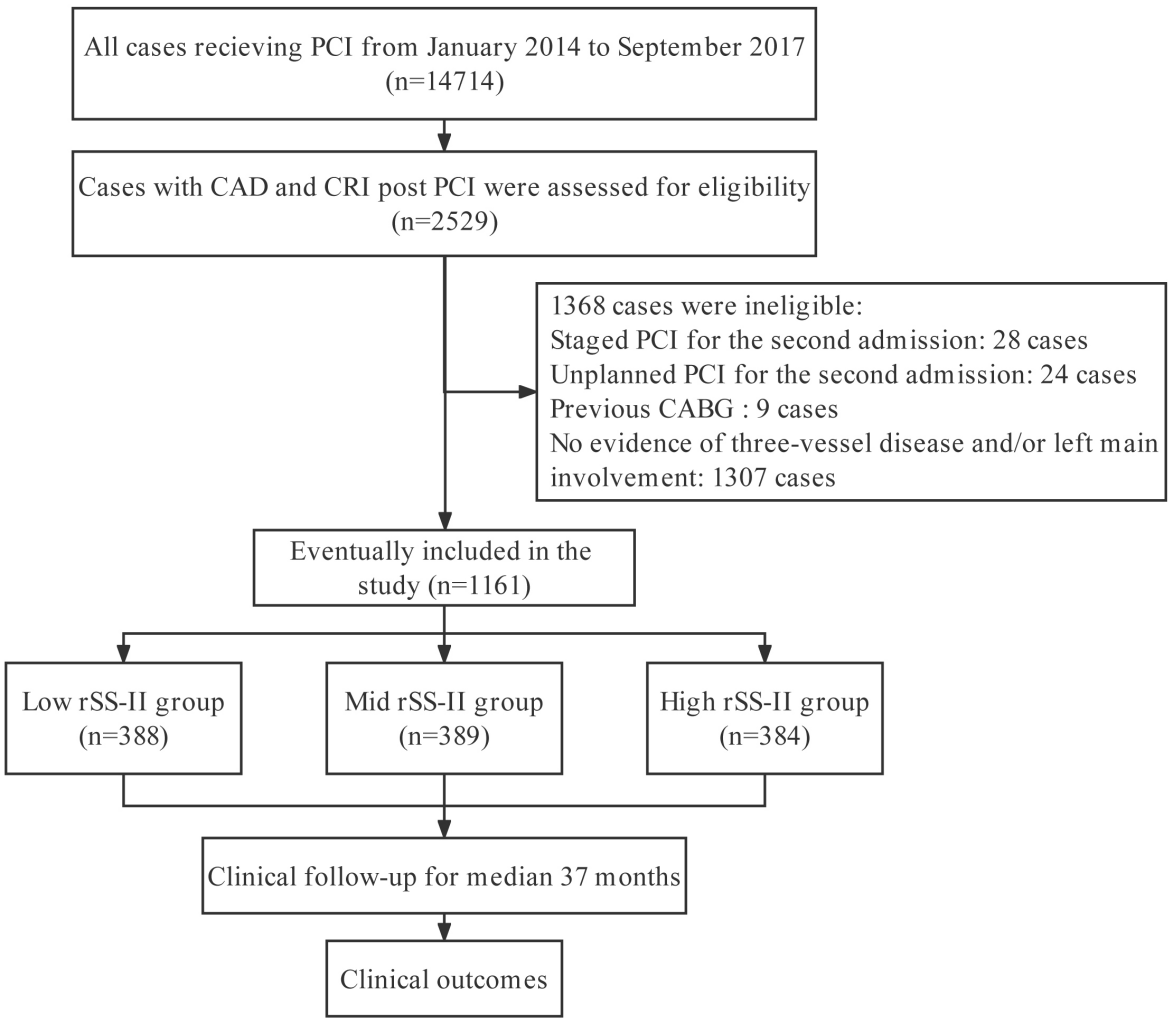
**Flowchart of study**. CABG, coronary artery bypass grafting; CAD, coronary artery disease; CRI, chronic renal insufficiency; PCI, percutaneous coronary intervention; rSS-II, residual SYNTAX (Synergy Between Percutaneous Coronary Intervention With Taxus and Cardiac Surgery) score II.

The design and methods of this study have been described in previous research [15]. Briefly, 2529 consecutive patients with CAD and CRI who received PCI at Cangzhou Central Hospital of Hebei Medical University were retrospectively screened from January 2014 to September 2017. Patients were excluded if they had previous coronary artery bypass grafting, staged PCI, or unplanned PCI during a second hospitalization. Additionally, 1312 patients with no evidence of three-vessel disease or left main disease involvement were excluded from the study. Ultimately, 1161 individuals with complex CAD, defined as three-vessel diseaseand/or left main disease (stenosis ≥50%), were selected to participate in this clinical research (Fig. 1). CRI was defined as an estimated glomerular filtration rate (eGFR) <90 mL/min per 1.73 m^2^, calculated using the simplified Modification of Diet in Renal Disease formula [18]. Based on the tertiles of the rSS-II, three patient groups were established: low-rSS-II (n = 388), medium-rSS-II (n = 389), and high-rSS-II groups (n = 384). To minimize bias, we enforced diagnostic criteria, trained staff, and applied rigorous statistical methods for accurate data analysis. The ethics committee of Cangzhou Central Hospital of Hebei Medical University approved this research, which was conducted in accordance with the principles of the Declaration of Helsinki. Written informed consent was obtained from all patients involved in the study.

### 2.2 Determination of SS-II, rSS, and rSS-II

The calculation of rSS was performed using the SS online calculator, which considers obstructive coronary stenosis after PCI. IR was defined as an rSS value greater than 0. To evaluate the SS-II for PCI, two anatomical variables (SS and ULMCA disease) and six clinical variables (age, creatinine clearance, LVEF, sex, COPD, and peripheral vascular disease) were included using the online calculator available at http://www.syntaxscore.org [13]. The rSS-II was determined as the post-PCI SS-II, calculated using the PCI SS-II calculator based on the online calculator.

### 2.3 Endpoints and Definitions

Follow-up assessments were conducted for all patients through telephone interviews or clinic visits, and clinical outcome data were verified by reviewing medical records. The primary endpoints of the study were all-cause mortality (ACM) and cardiac mortality (CM). In cases where a clear non-cardiac etiology was not identified, death was assumed to be due to a cardiac reason. The secondary endpoint was major adverse cardiovascular and cerebrovascular events (MACCEs), which was defined as a composite of ACM, myocardial infarction (MI), stroke, or unplanned revascularization. During the follow-up period, the fourth universal definition was used as the basis for defining MI [19].

### 2.4 Statistical Analysis

The Kolmogorov-Smirnov test was used to assess the normal distribution of continuous variables. All variables exhibited skewed distributions and were therefore represented as median (interquartile range, IQR). Comparative analysis of these variables was performed using the Kruskal-Wallis *H* test. Categorical variables were presented in terms of counts and proportions (%), and their comparative analysis was conducted using the Pearson χ^2^ test or Fisher’s exact test, depending on the appropriateness of each test. The Kaplan-Meier method and the Log-rank test were utilized to estimate and compare the cumulative rates of clinical events, respectively. Cox proportional hazard ratios (HR) were employed to make comparisons across three groups. Multivariate Cox regression analysis was performed to determine the significance of rSS-II in predicting long-term prognoses in a multivariate model, including variables from the univariate analysis with a *p*-value < 0.1. However, a few variables in the rSS-II, such as gender and eGFR, were excluded from the multivariate Cox regression analysis to avoid multicollinearity. The proportional hazards assumption was confirmed using the Schoenfeld residuals test, and no relevant violations were found.

The validation of the rSS-II was conducted according to the strategy proposed by Steyerberg and Vergouwe [20]. The discriminatory capacity of the rSS-II was assessed using the area under the curve (AUC) of time-dependent receiver operating characteristic (ROC) curves. The AUC values of the rSS-II were compared with those of the rSS and SS-II based on a previous study [21]. Calibration of the rSS-II, rSS, and SS-II was performed using the Greenwood-Nam-D’ Agostino goodness-of-fit test by comparing the observed and predicted outcomes [22]. A calibration plot of the predicted versus observed probabilities was developed, following recent statistical recommendations for developing and validating risk scores [23]. The calibrations of different models were compared at a median follow-up period of 37 months using the mean, median, and 90th percentile of absolute calibration error (integrated calibration index (ICI), E50, and E90, respectively) [24]. Decision curve analysis (DCA) was used to evaluate which model was most helpful in identifying individuals with a higher risk of long-term outcomes at 37 months [25]. Statistical significance was determined when a two-sided *p*-value was < 0.05. All statistical analyses were conducted using R software version 4.2.0 (R Foundation for Statistical Computing, Vienna, Austria), with the packages *survival*,* timeROC*, *survival.calib*, and* ggDCA*.

## 3. Results

### 3.1 Baseline Characteristics

The median age of the patients was 67.0 (61.0–71.0) years, and the body mass index (BMI) was 26.1 (24.8–27.3) kg/m^2^. Among the patients, 59.3% were men. Table 1 displays the patient demographics and clinical characteristics according to the tertiles of the rSS-II. Patients in the high-rSS-II category were older and had lower baseline characteristics such as male gender, serum creatinine, hemoglobin, eGFR, and LVEF, but had higher fasting glucose and total cholesterol levels (all *p* < 0.01) compared to those in the low- and medium-rSS-II groups. The prevalence of diabetes was higher and smoking was lower in the medium- and high-rSS-II groups compared to the low-rSS-II category (all *p* < 0.05). Additionally, patients in the high-rSS-II group had more complex baseline angiography and procedural characteristics, including bifurcation or trifurcation, severe tortuosity, stent count per patient, total stent length, and stent length >100 mm, compared to the low- and medium-rSS-II groups (all *p* < 0.05) (Table 2).

**Table 1.** **Clinical characteristics of three groups based on rSS-II**.

		Low (n = 388)	Medium (n = 389)	High (n = 384)	*p*-value
Age (years)		66.0 (55.0–66.0)	67.0 (62.0–71.0)	70.0 (67.0–75.0)	<0.001
Male (%)		372 (95.9)	216 (55.5)	101 (26.3)	<0.001
BMI (kg/m^2^)		26.4 (25.2–27.6)	26.1 (24.8–27.2)	25.8 (24.6–27.1)	<0.001
Risk factors (%)					
	Hypertension	255 (65.7)	271 (69.7)	277 (72.1)	0.150
	Dislipidemia	160 (41.2)	147 (37.8)	161 (41.9)	0.453
	Diabetes	78 (20.1)	105 (27.0)	122 (31.8)	0.001
	Smoking	57 (14.7)	46 (11.8)	30 (7.8)	0.011
Previous history (%)					
	Myocardial infarction	31 (8.0)	35 (9.0)	44 (11.5)	0.239
	PCI	46 (11.9)	43 (11.1)	43 (11.2)	0.932
	Stroke	36 (9.3)	42 (10.8)	51 (13.3)	0.203
Initial presentation					
	STEMI (%)	112 (28.9)	97 (24.9)	119 (31.0)	0.130
	Heart rate (beats/min)	73.0 (70.0–77.0)	73.0 (70.0–77.0)	73.0 (70.0–79.0)	0.932
	SBP (mmHg)	133.0 (120.0–145.0)	130.0 (124.0–149.0)	132.0 (120.0–150.0)	0.748
Renal and cardiac function					
	eGFR (mL/min per 1.73 m^2^)	83.3 (77.8–87.1)	78.5 (71.1–84.1)	68.0 (55.2–77.0)	<0.001
	LVEF (%)	63.0 (60.0–64.0)	62.0 (59.0–64.0)	61.0 (54.0–64.0)	<0.001
	LVEDD (mm)	48.0 (46.0–50.0)	47.0 (45.0–50.0)	47.0 (45.0–51.0)	0.001
Laboratory data					
	cTNI (ng/mL)	0.30 (0.05–1.80)	0.32 (0.05–1.34)	0.45 (0.05–2.09)	0.067
	CK-MB (U/L)	14.7 (12.0–23.3)	14.9 (11.9–22.0)	16.3 (12.1–30.4)	0.111
	Creatine (µmol/L)	86.0 (83.0–92.0)	83.0 (67.0–95.0)	81.0 (70.0–105.0)	<0.001
	Fasting glucose (mmol/L)	6.4 (5.3–8.4)	6.7 (5.5–9.1)	7.2 (5.8–10.0)	<0.001
	TC (mmol/L)	4.4 (3.9–4.9)	4.3 (3.9–5.0)	4.5 (4.1–5.2)	0.020
	Hemoglobin (g/L)	139.0 (130.0–149.0)	131.0 (120.0–141.0)	123.0 (113.0–132.0)	<0.001

Values are median (interquartile range) or n (%). BMI, body mass index; CK-MB, creatine kinase-MB; cTNI, cardiac troponin I; eGFR, estimated glomerular filtration rate; LVEDD, left ventricular end-diastolic diameter; LVEF, left ventricular ejection fraction; PCI, percutaneous coronary intervention; rSS-II, residual SYNTAX (Synergy Between Percutaneous Coronary Intervention With Taxus and Cardiac Surgery) score II; SBP, systolic blood pressure; STEMI, ST-elevation myocardial infarction; TC, total cholesterol.

**Table 2.** **Angiographic and procedural characteristics**.

		Low (n = 388)	Medium (n = 389)	High (n = 384)	*p*-value
Diseased vessels (%)					<0.001
	3-vessel	330 (85.1)	339 (87.1)	333 (86.7)	
Left main					
	Isolated	2 (0.5)	1 (0.3)	0	
	Plus-1-vessel	6 (1.5)	3 (0.8)	1 (0.3)	
	Plus-2-vessel	23 (5.9)	22 (5.7)	6 (1.6)	
	Plus-3-vessel	27 (7.0)	24 (6.2)	44 (11.5)	
Lesion anatomical characteristics					
	Lesion length >20 mm	278 (71.7)	283 (72.8)	286 (74.5)	0.672
	Bifurcation or trifuraction	64 (16.5)	69 (17.7)	45 (11.7)	0.049
	Aorto-ostial lesion	3 (0.8)	6 (1.5)	10 (2.6)	0.132
	Heavy calcification	16 (4.1)	19 (4.9)	28 (7.3)	0.128
	Severe tortuosity	6 (1.6)	12 (3.1)	18 (4.7)	0.042
	Thrombus	18 (4.6)	26 (6.7)	28 (7.3)	0.277
	CTO	34 (8.8)	30 (7.7)	35 (9.1)	0.768
Location of target vessels					
	LM	41 (10.6)	21 (5.4)	9 (2.3)	<0.001
	LAD	229 (59.0)	213 (54.8)	158 (41.2)	<0.001
	LCX	156 (40.2)	131 (33.7)	121 (31.5)	0.031
	RCA	196 (50.5)	183 (47.0)	202 (52.6)	0.295
Procedural characteristics					
	Stent per patient	2.0 (1.0–3.0)	2.0 (1.0–3.0)	2.0 (1.0–2.0)	<0.001
	Total length of the stent (mm)	56.0 (35.0–86.0)	50.0 (33.0–72.0)	46.0 (29.0–66.0)	<0.001
	Stent length >100 mm (%)	54 (13.9)	42 (10.8)	26 (6.8)	0.005
	CR (%)	25 (6.4)	10 (2.6)	4 (1.0)	<0.001
	Mean stent diameter (mm)	3.0 (2.8–3.2)	2.9 (2.7–3.1)	2.8 (2.7–3.0)	0.001
	Minimum stent diameter (mm)	2.8 (2.5–3.0)	2.8 (2.5–3.0)	2.8 (2.5–3.0)	0.488
	Maximum stent diameter (mm)	3.0 (3.0–3.5)	3.0 (2.8–3.5)	3.0 (2.8–3.5)	<0.001

Values are median (interquartile range) or n (%). CR, complete revascularization; CTO, chronic total occlusions; LAD, left anterior descending artery; LCX, left circumflex; LM, left main; RCA, right coronary artery.

### 3.2 Prognostic Value of rSS-II

**Fig. 2.**
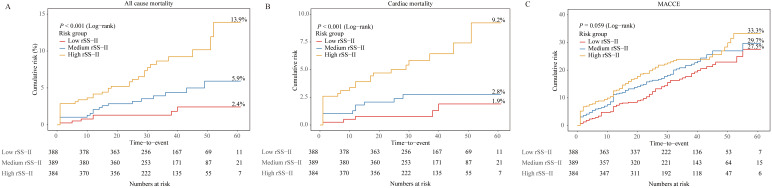
**Kaplan-Meier cumulative risk curves for clinical outcomes according to the rSS-II tertiles**. Kaplan-Meier cumulative risk curves for all-cause mortality (A), cardiac mortality (B), and MACCE (C). MACCE, major adverse cardiovascular and cerebrovascular event; SYNTAX, Synergy Between Percutaneous Coronary Intervention With Taxus and Cardiac Surgery; rSS-II, residual SYNTAX score II.

The median follow-up time for the patients was 37 months (range: 19–61 months). During this period, ACM, CM, and MACCE occurred in 57 (4.9%), 39 (3.4%), and 248 (21.4%) patients, respectively. The incidence of ACM and CM was significantly higher in the high-rSS-II group compared to the medium- and low-rSS-II groups (ACM: 13.9% vs. 5.9% vs. 2.4%, *p* < 0.001; CM: 9.2% vs. 2.8% vs. 1.9%, *p* < 0.001). However, there was no significant difference in the prevalence of MACCE among the three groups (33.3% vs. 29.7% vs. 27.5%, *p* = 0.059) (Fig. 2).

The univariate Cox regression analysis findings are presented in **Supplementary Table 1**. The high-rSS-II group had significantly higher risks for ACM (2.12- and 5.08-fold) and CM (2.57- and 5.09-fold) compared to the medium- and low-rSS-II groups, respectively (all *p* < 0.05). However, the rSS-II only discriminated between individuals in the high-rSS-II group and the low-rSS-II group for MACCE (*p* = 0.017) (**Supplementary Fig. 1**). Multivariate Cox regression analysis showed that rSS-II was an independent predictor of ACM (HR 1.08 [95% CI: 1.04–1.12], *p* < 0.001) and CM (HR 1.07 [95% CI: 1.02–1.12], *p* = 0.009 (Table 3).

**Table 3.** **Multivariable Cox regression analysis of long-term outcomes**.

Variables		HR (95% CI)	*p*-value
All-cause mortality			
	Hypertension	0.57 (0.33–0.96)	0.035
	Dislipidemia	0.54 (0.29–0.99)	0.047
	rSS-II	1.08 (1.04–1.12)	<0.001
Cardiac mortality			
	Dislipidemia	0.44 (0.21–0.92)	0.029
	Previous MI	2.52 (1.11–5.70)	0.027
	rSS-II	1.07 (1.02–1.12)	0.009
MACCE			
	Previous PCI	1.45 (1.01–2.07)	0.042

MACCE, major adverse cardiovascular and cerebrovascular event; MI, myocardial infarction; PCI, percutaneous coronary intervention; rSS-II, residual SYNTAX (Synergy Between Percutaneous Coronary Intervention With Taxus and Cardiac Surgery) score II; HR, hazard ratio.

### 3.3 Discrimination Power

**Fig. 3.**
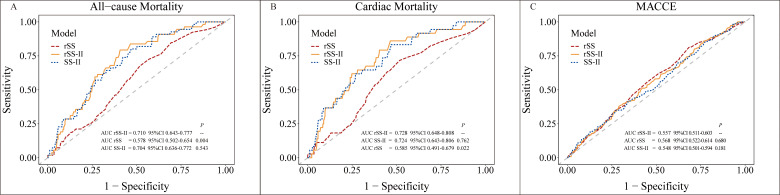
**ROC curve for the discrimination capability of rSS-II compared with rSS and SS-II**. Time-dependent receiver operating characteristic (ROC) curves for (A) all-cause mortality; (B) cardiac mortality; and (C) MACCE based on rSS, SS-II, and rSS-II are shown. AUC, the area under the curve; MACCE, major adverse cardiovascular and cerebrovascular event; SYNTAX, Synergy Between Percutaneous Coronary Intervention With Taxus and Cardiac Surgery; rSS-II, residual SYNTAX score II; rSS, residual SYNTAX score; SS-II, SYNTAX score II.

The AUCs of time-dependent ROC at 37 months for rSS-II, rSS, and SS-II were calculated and compared (Fig. 3). The discrimination capability of rSS-II was acceptable for predicting ACM and CM. The AUCs of rSS-II for ACM (0.710 vs. 0.578, *p* = 0.004) and CM (0.728 vs. 0.585, *p* = 0.022) were significantly higher than those of rSS. The AUCs of rSS-II had an increasing trend compared to SS-II (AUC: 0.704 for ACM; 0.724 for CM), but the difference was not statistically significant (all *p* > 0.05). However, the discrimination capability of rSS-II was poor for predicting MACCE. The AUC of rSS-II was 0.557, which was not significantly different from those of rSS (AUC: 0.568) and SS-II (AUC: 0.548) (all *p* > 0.05).

### 3.4 Calibration

**Fig. 4.**
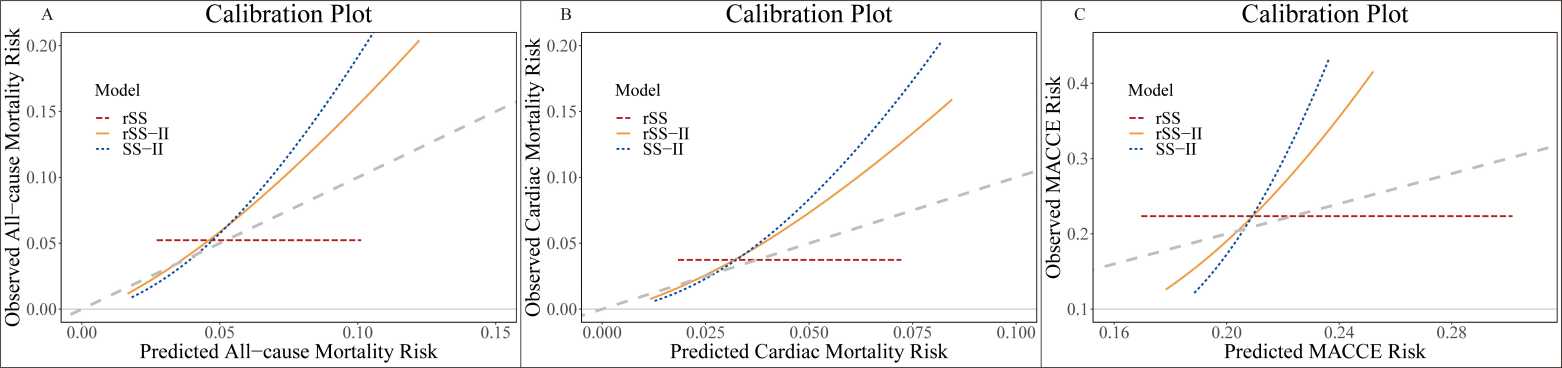
**Calibration plot of predicted vs. observed outcomes**. Calibration plots for (A) all-cause mortality, (B) cardiac mortality, and (C) MACCE based on rSS, SS-II, and rSS-II are shown. MACCE, major adverse cardiovascular and cerebrovascular event; SYNTAX, Synergy Between Percutaneous Coronary Intervention With Taxus and Cardiac Surgery; rSS-II, residual SYNTAX score II; rSS, residual SYNTAX score; SS-II, SYNTAX score II.

The calibration curve of rSS-II for the risk of clinical outcomes showed the best agreement between prediction and observation (Fig. 4). The Greenwood-Nam-D’Agostino test revealed that all three models were well-calibrated for predicting ACM, CM, and MACCE (all *p* > 0.05) (**Supplementary Table 2**). The calibration metrics values for the three risk models are shown in **Supplementary Table 3**. Compared to rSS and SS-II, rSS-II showed better calibration for ACM, with ICI = 0.01, E50 = 0.0037, and E90 = 0.0228. However, rSS had the worst calibration for ACM, with ICI = 0.0145, E50 = 0.0136, and E90 = 0.0239. For CM and MACCE, rSS-II displayed acceptable calibration compared to rSS and SS-II.

### 3.5 Decision Curve Analysis

The net benefit of rSS, SS-II, and rSS-II at 37 months was evaluated using DCA for the examined prognosis probabilities (**Supplementary Fig. 2**). The rSS-II showed a high net benefit across a wide range of threshold probabilities for predicting ACM (1.1%–10.7% and 22.2%–28.3%) and CM (0.6%–8.0% and 17.9%–21.5%). However, SS-II had a limited range of threshold probabilities, and rSS showed a low net benefit. Additionally, rSS-II had an acceptable net benefit across a wide range of threshold probabilities for predicting MACCE (14.6%–22.7% and 32.6%–34.1%).

## 4. Discussion

Several important findings were observed in this study: (1) rSS-II was able to predict long-term prognosis over a median follow-up time of 37 months in individuals with complex CAD and CRI after PCI. (2) Compared to rSS and SS-II, rSS-II showed improved calibration, higher net benefit for clinical performance, and better discriminative capacity. (3) rSS-II was able to independently predict long-term prognoses and effectively stratify patients based on their risks of ACM and CM after PCI in individuals with complex CAD and CRI.

Despite Généreux* et al*.’s [2] claim that post-PCI residual coronary stenosis is a nidus of new events, the treatment of non-culprit vessels and the timing of PCI have been questionable until recently. The SYNTAX trial showed that IR has a negative effect on long-term clinical outcomes and is a surrogate biomarker of significant coronary complexity and clinical comorbidity [26]. The COMPLETE trial reported that CR during the index hospitalization is linked to a clear clinical benefit compared to culprit-lesion-only PCI in individuals with STEMI and multivessel CAD [4]. Several studies have also shown that CR improves the prognosis of individuals receiving PCI for ACS with CKD [7, 26]. However, in the CKD population, Kim* et al*. [27] found no therapeutic benefit of angiographic CR compared to IR. In this study, first-generation DES was used by about 60% of patients, while second-generation DES has been reported to have a protective impact on clinical outcomes in CKD-affected individuals [28].

The Grand-DES investigation found that CKD-affected individuals who underwent multivessel PCI during the index hospitalization had a reduced incidence of death and cardiovascular events for up to three years after the procedure [7]. However, due to high adverse event rates and low procedural success rates, only a small proportion (3.4%) of patients received CR in this study. The findings of this research are consistent with those reported by the IR group of the trial, indicating that a higher residual disease burden, equivalent to a higher rSS-II, increases unfavorable cardiovascular outcomes in individuals with complex CAD and CRI after PCI.

A previous study demonstrated that rSS, which quantifies the residual burden of anatomical coronary disease, evaluated the predictive value of IR after PCI [2]. Farooq* et al*. [3] indicated that rSS was capable of independently predicting 5-year ACM in individuals affected by complex CAD after PCI using drug-eluting stents in the SYNTAX trial. However, the application of rSS in clinical practices is limited due to the absence of clinical variables, which has led to the establishment and validation of rSS-II for predicting the prognosis of individuals receiving PCI. Boukhris* et al*. [29] demonstrated that rSS-II could anticipate midterm outcomes in patients with complex ACS. Bortnick* et al*. [16] further suggested that rSS-II was associated with long-term ACM using Cox regression analysis in a post-STEMI cohort from the Montefiore STEMI registry (HR: 2.46, 95% CI: 1.51–3.99, *p* < 0.001), which is consistent with the findings of this research. However, the main distinction is that the registry did not stratify different risk outcomes based on rSS-II for patients with STEMI. Risk stratification is important for identifying individuals at a higher risk of a particular health condition in order to offer proven interventions. In comparison, patients in the SHINANO registry were classified into two categories based on the cut-off value calculated using ROC analysis for ACM [17], showing that the incidence of ACM in the high-score group was significantly higher than in the low-score group (38.0% vs. 5.7%, *p* < 0.01). In this research, patients were classified into three categories based on rSS-II. Compared to the low- and medium-rSS-II groups, the high-rSS-II group had a significantly higher incidence of ACM and CM.

In the SHINANO registry, Kashiwagi* et al*. [17] indicated that rSS-II might be a crucial tool in predicting long-term mortality in individuals with ACS and multivessel disease following PCI. The AUC of rSS-II for predicting ACM (AUC = 0.82, 95% CI: 0.74–0.91) was significantly better compared to that of rSS (AUC = 0.54, 95% CI: 0.1–0.67) (*p* < 0.001). Although the AUC of rSS-II showed an increasing trend compared to SS II (0.82 [0.74–0.91] vs. 0.80 [0.71–0.90], *p* = 0.089), it was not statistically significant. Furthermore, this research demonstrated a similar discriminative power of rSS-II compared to the original rSS and SS-II in predicting ACM in individuals with complex CAD and CRI following PCI. However, the main differences existed in the sub analysis performed using data from the SHINANO registry, which recruited only 120 patients, and the validation of rSS-II was based solely on its discriminative power, lacking calibration and evaluation of clinical utility.

Discrimination, calibration, and clinical utility are well-established components of a predictive model. Steyerberg* et al*. [30] emphasized that decision-analytic measures should be reported prior to applying the prediction model for clinical decision-making. This approach can potentially assist physicians in evaluating the significance of information by using a risk assessment tool to compare potential risks and benefits. In this research, the advantage of rSS-II over rSS and SS-II for clinical applications was determined through DCA, given that rSS and SS-II were already being used in clinical research and practices. The rSS-II demonstrated acceptable net benefits across various threshold probabilities for long-term prognoses compared to rSS and SS-II.

## 5. Limitations

There are certain limitations to this study. Firstly, it was a retrospective, single-center study. Therefore, further prospective studies and multicenter datasets are necessary to assess the generalizability and validity of rSS-II. Secondly, pre-PCI creatinine clearance and LVEF were used in the study. However, a previous study reported that creatinine clearance and left LVEF in SS II should be modified using PCI [29] and used as post-PCI in rSS-II. Hence, further studies including postoperative creatinine clearance and LVEF are needed to validate the performance of rSS-II in predicting the prognosis of individuals with complex CAD and CRI after PCI. Finally, rSS-II was validated only in the Chinese population. Therefore, additional studies with larger population samples are required to further validate its performance.

## 6. Conclusions

The present study provides favorable evidence for the precise use of rSS-II in predicting long-term clinical outcomes in individuals with complex CAD and CRI after PCI. Over a median follow-up of 37 months, rSS-II demonstrated good discriminatory power for risk prediction of ischemic outcomes. Furthermore, it can identify patients who may benefit from further revascularization.

## Availability of Data and Materials

The datasets used and/or analyzed during the current study are available from the corresponding author on reasonable request.

## Supplementary Material

Supplementary material associated with this article can be found, in the online version, at https://doi.org/10.31083/RCM26962.
