# Supplementary material for: Predictive Value of Residual SYNTAX Score II for Patients With Complex Coronary Disease and Chronic Renal Insufficiency After Percutaneous Coronary Intervention
Source: Rev Cardiovasc Med. 2025 May 27;26(5):26962. doi: 10.31083/RCM26962 (PMC12135670; doi:10.31083/RCM26962)
Supplement: Supplementary file 1 [file 2153-8174-26-5-26962-s1.docx]

Table S1 Univariable Cox regression analysis of long-term outcomes

| Variables | All-cause Mortality | | Cardiac Mortality | | MACCE | |
| --- | --- | --- | --- | --- | --- | --- |
|  | HR (95%CI) | p-value | HR (95%CI) | p-value | HR (95%CI) | p-value |
| Age | 1.06(1.02-1.10) | 0.001 | 1.05(1.00-1.09) | 0.029 | 1.01(0.99-1.02) | 0.401 |
| Male | 1.11(0.66-1.87) | 0.698 | 0.99(0.53-1.88) | 0.986 | 0.79(0.61-1.02) | 0.067 |
| BMI | 0.88(0.79-0.99) | 0.032^*^ | 0.90(0.78-1.03) | 0.134 | 1.02(0.97-1.08) | 0.394 |
| Hypertension | 0.50(0.30-0.85) | 0.010^*^ | 0.65(0.34-1.22) | 0.181^*^ | 1.09(0.83-1.43) | 0.538 |
| Dislipidemia | 0.51(0.28-0.92) | 0.025^*^ | 0.50(0.24-1.02) | 0.057^*^ | 0.91(0.70-1.17) | 0.463 |
| Diabetes | 1.20(0.68-2.12) | 0.525 | 1.76(0.92-3.36) | 0.086 | 1.24(0.94-1.62) | 0.124 |
| Smoking | 1.12(0.51-2.47) | 0.778 | 1.17(0.46-3.00) | 0.739 | 0.75(0.48-1.16) | 0.194 |
| Previous MI | 2.10(1.06-4.15) | 0.034^*^ | 2.52(1.16-5.49) | 0.020^*^ | 1.28(0.86-1.89) | 0.222 |
| Previous PCI | 0.93(0.40-2.16) | 0.865 | 1.43(0.60-3.41) | 0.420 | 1.40(0.99-2.00) | 0.061^*^ |
| Previous Stroke | 0.27(0.07-1.11) | 0.070^*^ | 0.41(0.10-1.70) | 0.218 | 1.17(0.81-1.69) | 0.409 |
| Heart rate | 0.99(0.96-1.01) | 0.349 | 0.98(0.95-1.01) | 0.156 | 1.00(0.99-1.01) | 0.852 |
| SBP | 1.00(0.98-1.01) | 0.447 | 0.99(0.98-1.01) | 0.453 | 1.00(1.00-1.01) | 0.419 |
| eGFR | 0.98(0.96-0.99) | 0.001 | 0.97(0.95-0.98) | <0.001 | 0.99(0.98-0.99) | 0.001 |
| LVEF | 0.96(0.94-0.99) | 0.003 | 0.96(0.93-1.00) | 0.024 | 0.98(0.97-1.00) | 0.009 |
| LVEDD | 1.05(0.99-1.10) | 0.113 | 1.08(1.02-1.15) | 0.010^*^ | 1.03(1.00-1.06) | 0.025^*^ |
| cTNI | 1.04(1.02-1.06) | <0.001^*^ | 1.05(1.03-1.07) | <0.001^*^ | 1.02(1.01-1.04) | <0.001^*^ |
| CK-MB | 1.00(1.00-1.01) | <0.001^*^ | 1.00(1.00-1.01) | <0.001^*^ | 1.00(1.00-1.00) | <0.001^*^ |
| Creatine | 1.01(1.00-1.01) | 0.057 | 1.01(1.00-1.01) | 0.005 | 1.01(1.00-1.01) | <0.001 |
| Fasting glucose | 1.06(1.02-1.10) | 0.004^*^ | 1.08(1.03-1.12) | <0.001^*^ | 1.03(1.00-1.06) | 0.040^*^ |
| TC | 0.88(0.68-1.14) | 0.339 | 0.86(0.62-1.18) | 0.352 | 0.98(0.86-1.10) | 0.685 |
| Hemoglobin | 0.99(0.97-1.00) | 0.106 | 0.98(0.96-1.00) | 0.015^*^ | 1.00(0.99-1.01) | 0.810 |
| rSS-II | 1.08(1.04-1.12) | <0.001^*^ | 1.08(1.04-1.13) | <0.001^*^ | 1.02(1.00-1.04) | 0.019^*^ |
| rSS-II indicates residual SYNTAX (Synergy Between Percutaneous Coronary Intervention With Taxus and Cardiac Surgery) score II; BMI, body mass index; PCI, percutaneous coronary intervention; SBP, Systolic blood pressure; STEMI, ST elevation myocardial infarction; eGFR, estimated glomerular filtration rate; LVEF, left ventricular ejection fraction; LVEDD, left ventricular end-diastolic diameter; TC, total cholesterol; MACCE, major adverse cardiovascular and cerebrovascular events. | | | | | | |

Table S2 Greenwood-Nam-D'Agostino fit test

| Clinical Outcomes | rSS | | rSS-II | | SS-II | |
| --- | --- | --- | --- | --- | --- | --- |
|  | χ^2^ | P value | χ^2^ | P value | χ^2^ | P value |
| All-cause Death | 3.60 | 0.463 | 3.95 | 0.557 | 4.01 | 0.548 |
| Cardiac Death | 0.47 | 0.491 | 2.60 | 0.761 | 4.32 | 0.634 |
| MACCE | 5.84 | 0.558 | 3.39 | 0.758 | 2.66 | 0.850 |
| MACCE indicates major adverse cardiovascular and cerebrovascular events; rSS indicates residual SYNTAX score; rSS-II; residual SYNTAX score II; SS-II, SYNTAX score II; SYNTAX, Synergy Between Percutaneous Coronary Intervention With Taxus and Cardiac Surgery. | | | | | | |

Table S3 Calibration metrics between different prediction methods for clinical outcomes

| Calibration Metrics | rSS | rSS-II | SS-II |
| --- | --- | --- | --- |
| All-cause Death | | | |
| ICI | 0.015 | 0.010 | 0.014 |
| E50 | 0.014 | 0.004 | 0.007 |
| E90 | 0.024 | 0.023 | 0.027 |
| Cardiac Death | | | |
| ICI | 0.011 | 0.009 | 0.012 |
| E50 | 0.011 | 0.003 | 0.005 |
| E90 | 0.017 | 0.022 | 0.028 |
| MACCE | | | |
| ICI | 0.028 | 0.035 | 0.039 |
| E50 | 0.025 | 0.024 | 0.027 |
| E90 | 0.047 | 0.072 | 0.079 |
| ICI indicates integrated calibration index; E50, the median of absolute calibration error; E90, the 90th percentile of absolute calibration error; MACCE, major adverse cardiovascular and cerebrovascular events. | | | |


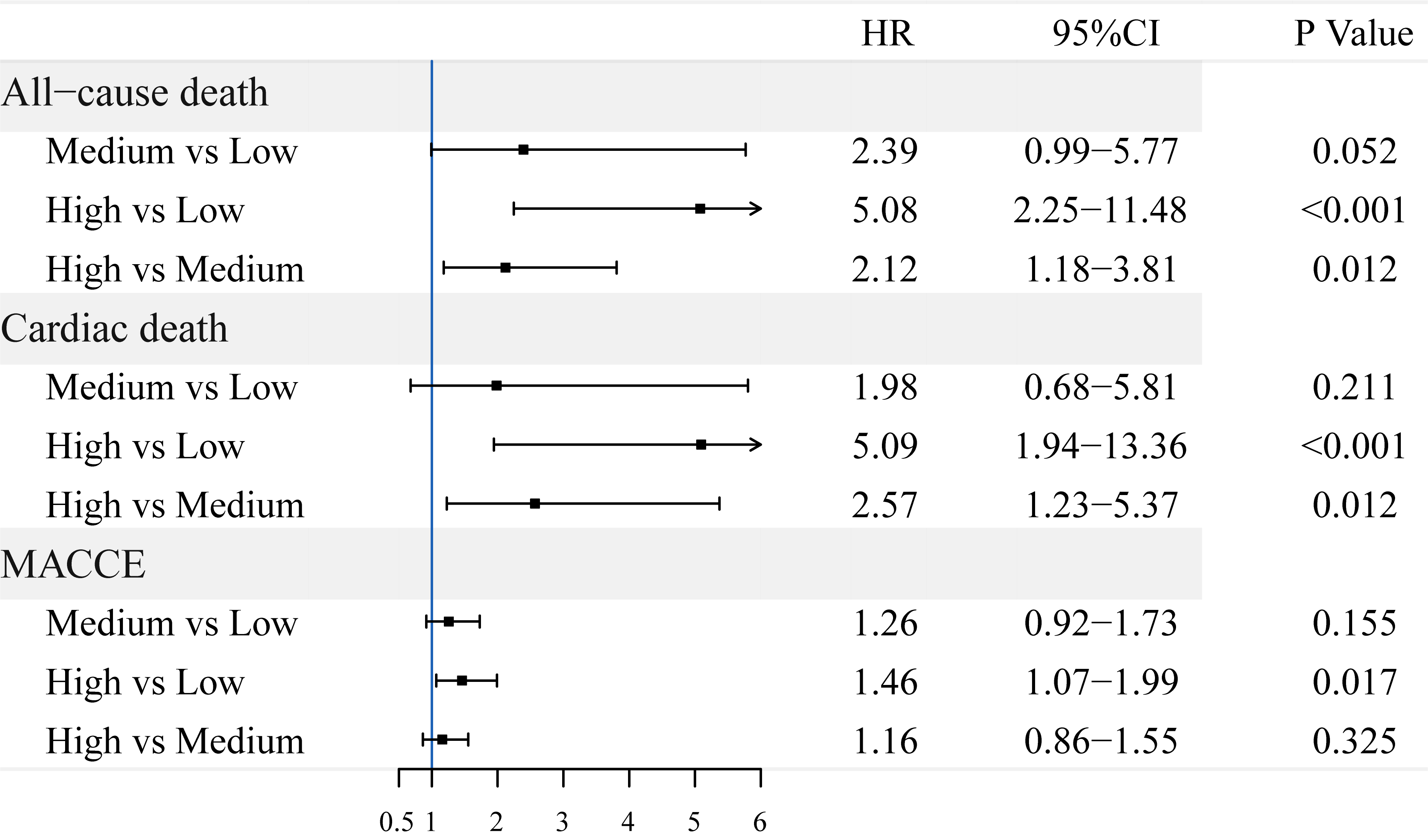


Figure S1 Forest plot for clinical outcomes according to the residual SYNTAX score II (rSS-II) tertiles.

MACCE indicates major adverse cardiovascular and cerebrovascular events; and SYNTAX, Synergy Between Percutaneous Coronary Intervention With Taxus and Cardiac Surgery.


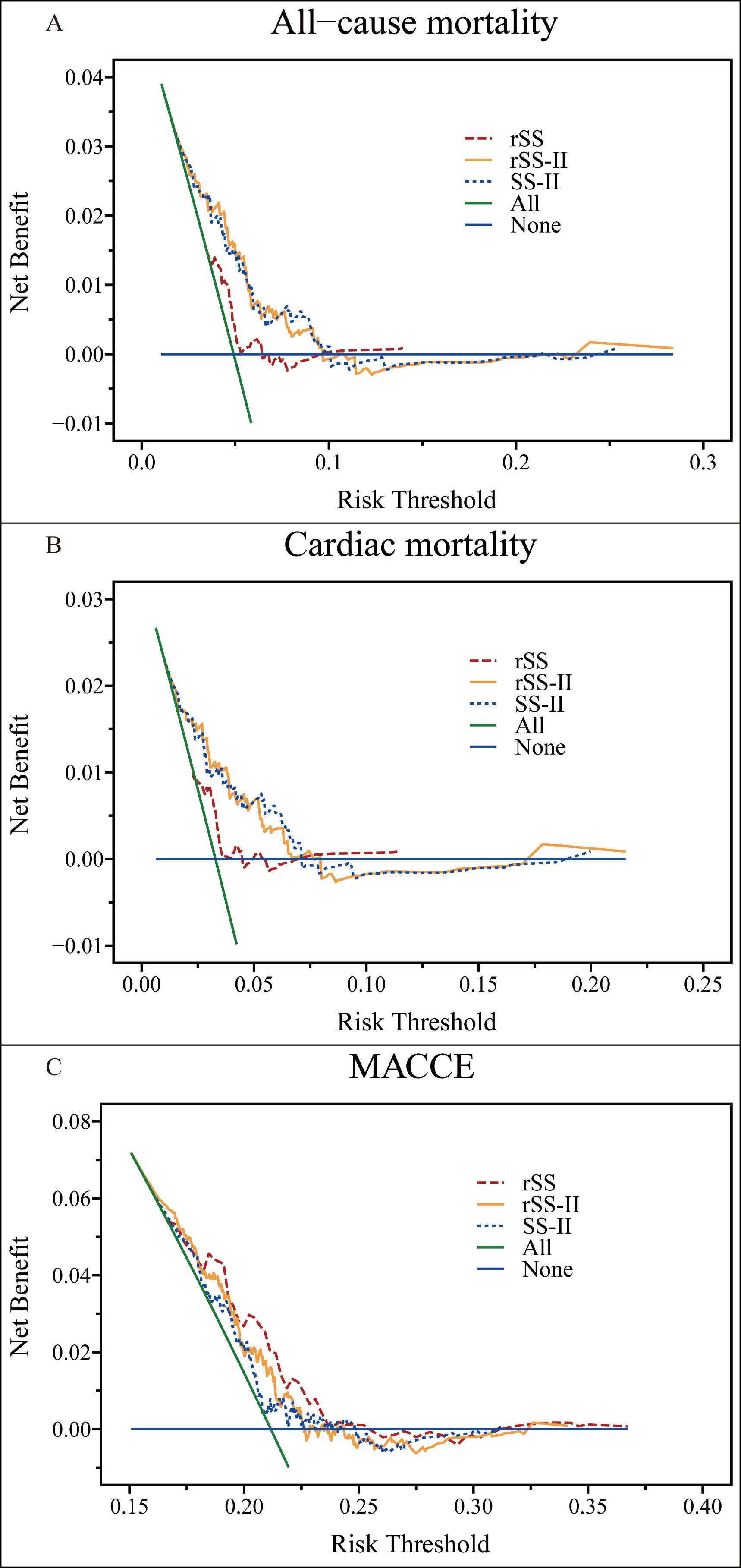


Figure S2 Decision curve analysis for residual SYNTAX score (rSS), SYNTAX score II (SS-II), and residual SYNTAX score II (rSS-II) in predicting (A) all-cause mortality, (B) cardiac mortality, and (C) MACCE.

The decision curve analysis graphically shows the clinical usefulness of rSS, SS-II, and rSS-II based on a continuum of potential thresholds for clinical outcomes risk (x axis) and the net benefit of using different models to risk stratify patients (y axis). Net benefit=true positive rate - (false positive rate × weighting factor), Weighting factor=Threshold probability/(1-threshold probability). MACCE indicates major adverse cardiovascular and cerebrovascular events; and SYNTAX, Synergy Between Percutaneous Coronary Intervention With Taxus and Cardiac Surgery.
